# Australian Healthcare Professionals’ Knowledge of and Attitudes toward Binge Eating Disorder

**DOI:** 10.3389/fpsyg.2017.01291

**Published:** 2017-08-07

**Authors:** Belinda Cain, Kimberly Buck, Matthew Fuller-Tyszkiewicz, Isabel Krug

**Affiliations:** ^1^Melbourne School of Psychological Sciences, The University of Melbourne, Melbourne VIC, Australia; ^2^School of Psychology, Deakin University, Burwood VIC, Australia; ^3^Centre for Social and Early Emotional Development, Deakin University, Burwood VIC, Australia

**Keywords:** eating disorders, binge eating disorder, obesity, knowledge, attitudes, diagnosis, treatment, weight bias

## Abstract

**Objective:** This study aimed to investigate Australian healthcare practitioners’ knowledge and attitudes toward binge eating disorder (BED).

**Method:** Participants were 175 healthcare professionals, who were randomized to one of two conditions that assessed diagnostic and treatment knowledge of either comorbid BED and obesity or only obesity via case vignette, as well as weight bias toward obese patients.

**Results:** Results suggested that participants demonstrated a reluctance to diagnose comorbid BED and obesity, that their knowledge of physical complications associated with BED was limited, and that they indicated a narrow range of evidence-based treatment options. When compared with levels of weight bias expressed by healthcare professionals in previous international studies, Australian clinicians were significantly less biased, however, still largely endorsed ‘negative’ attitudes toward obesity.

**Conclusion:** Findings suggest that future clinical training in eating disorders should therefore focus not only on diagnostic criteria, physical complications and treatment options, but also on practitioner attitudes toward eating and weight.

## Introduction

Although individuals with eating disorders (EDs), including anorexia nervosa (AN), bulimia nervosa (BN), and binge eating disorder (BED), are frequent users of the healthcare system, EDs often remain undiagnosed or inadequately treated (Cachelin et al., 2000; Simon et al., 2005). Potential reasons for deficits in the detection and treatment of AN and BN that have been identified in the literature include inadequate practitioner mental health literacy (MHL; e.g., Currin et al., 2009; Jones et al., 2013) and negative practitioner attitudes toward ED patients (e.g., Thompson-Brenner et al., 2012). Although obstacles to the diagnosis and treatment of BED have yet to be adequately explored, similar factors are likely to contribute to poor detection and intervention rates. However, because BED is also highly comorbid with obesity (Mitchell, 2016), known barriers to diagnosing and treating obesity may also be relevant, including stigmatizing attitudes, or ‘weight bias’ (e.g., Puhl et al., 2014). Using a case vignette methodology, this study aimed to explore potential obstacles that might be encountered by Australian healthcare professionals when diagnosing and treating patients with BED.

Binge eating disorder is a serious mental disorder that confers high levels of distress, functional impairment and severe physical complications (APA, 2013). It is the most prevalent ED; 3-month prevalence rates in a population of Australians over the age of 15 years for BED and subthreshold BED were 5.6 and 6.9%, respectively, compared with the 3-month prevalence of AN and BN which were both under 1% (Hay et al., 2015). BED was recognized in the fourth edition of the Diagnostic and Statistical Manual of Mental Disorders (DSM-IV; APA, 1994) under the diagnosis Eating Disorders Not Otherwise Specified (EDNOS), and was formally added to DSM-5 as a separate ED diagnosis (APA, 2013).

The majority of individuals with BED are overweight or obese and approximately 50% of BED sufferers are clinically obese (Montano et al., 2016). As a result of the frequent comorbidity with obesity, individuals with BED are at increased risk of medical complications associated with obesity, such as type II diabetes, cardiovascular disorders, and fertility issues (Sheehan and Herman, 2015). It is often these physical complications for which individuals with BED seek professional medical help, perhaps because they are more salient to sufferers than disordered eating behaviors, which individuals may be ambivalent about changing (Montano et al., 2016). Generally, help is sought from general practitioners (GPs)/physicians, psychiatrists, dietitians, and mental health professionals (Mond et al., 2007; Fursland and Watson, 2014). These medical and allied health professionals are collectively referred to as ‘frontline’ practitioners in this study.

Although individuals with EDs, including BED, are prolific users of the healthcare system for a range of physical complaints, EDs remain largely undetected (Hay et al., 2007). Low detection rates are highly problematic, resulting in the missed opportunity for early intervention, which can negatively affect prognosis (van Son et al., 2010).

**FIGURE 1:**
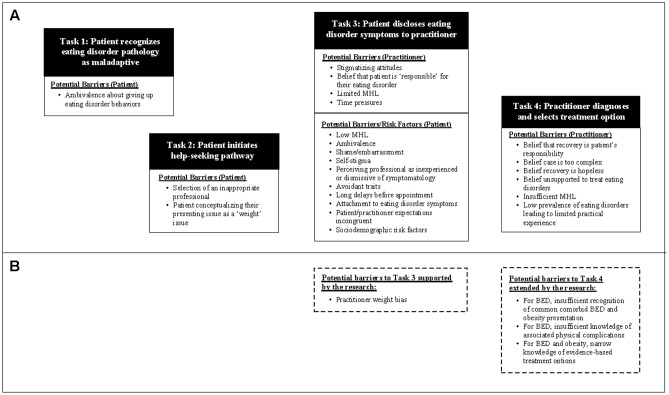
**(A)** Depiction of the help-seeking pathway based on barriers to diagnosis and treatment of EDs previously identified in the literature; **(B)** Barriers to Tasks 3 and 4 that were supported or extended by the present study.

The potential reasons for deficits in diagnoses have been explored largely in relation to AN and BN, to the exclusion of BED. Based on the ED literature to date (Mond et al., 2007; Evans et al., 2011), a sequence of tasks involving both the patient and treating healthcare professional must be achieved in order for an ED to be accurately diagnosed and managed. These tasks can be conceptualized as a ‘help-seeking pathway,’ involving four sequential steps: (1) Patient recognizes ED pathology as maladaptive; (2) Patient initiates helps-seeking pathway; (3) Patient discloses ED symptomology to healthcare provider; (4) Healthcare provider diagnoses ED and selects evidence-based treatment (**Figure 1A**). At each of these points, possible barriers have been identified within the literature that may prevent or delay an individual from moving to the next task in the sequence (e.g., Cachelin et al., 2000).

The help-seeking literature initially focused on why individuals do not seek help for disordered eating (i.e., barriers at the first two tasks of the help-seeking pathway; e.g., Cachelin et al., 2001; Cachelin and Striegel-Moore, 2006). However, a study conducted by Cachelin et al. (2000) suggested that in a sample of 61 women with a diagnosable ED, 85.2% wanted treatment, 57% reporting making contact with a health service, and 8% received treatment specifically for their ED. The implication is that whilst barriers do exist at the initial patient-driven tasks, many sufferers make it to task three (disclosure of symptoms to a healthcare provider), yet rarely receive effective treatment (Mond et al., 2007). It is thus likely that there are barriers at the level of the healthcare practitioner that may result in a lack of detection or intervention. For example, practitioners may have limited diagnostic knowledge or hold inaccurate beliefs about people with EDs, which may lead to symptoms of an ED being overlooked or misclassified.

Inadequate practitioner MHL, namely, the “knowledge and beliefs about mental disorders, which aid their recognition, management or prevention” (Jorm et al., 2000, p. 397), may contribute to the low detection and treatment rates for EDs. Research investigating medical professionals’ MHL of EDs has predominantly focused on AN and BN, with findings consistently highlighting substantial gaps in diagnostic and treatment knowledge (Currin et al., 2007; Hay et al., 2008; Jones et al., 2013). To date, only one study has explored GP knowledge of binge eating (Crow et al., 2004), with findings suggesting that physicians frequently do not screen for binge eating in populations of obese patients. Where comorbid binge eating and obesity was identified by GPs in the study, only a minority were aware of the need for additional monitoring and specialized treatment as compared with obesity alone. However, as this study was conducted prior to the introduction of BED as a formal diagnosis in DSM-5 (APA, 2013), practitioner MHL using current BED diagnostic criteria is largely unknown. In addition, the majority of MHL research in relation to EDs has been conducted internationally and has generally been limited to exploring the knowledge of GPs (e.g., Currin et al., 2007, 2009) or perceived practitioner MHL from the perspective of the patient (e.g., Räisänen and Hunt, 2014; Gulliksen et al., 2015). Further research is thus required that explores a range of frontline healthcare professionals’ MHL of BED, particularly in the Australian context.

Treatment outcomes are unlikely to be solely attributable to medical professionals’ knowledge: professionals’ perceptions of the disorder are also likely to affect therapeutic alliance, treatment options and outcome (Thompson-Brenner et al., 2012). Previous research has suggested that medical professionals may hold stigmatizing (negative stereotypical) attitudes toward individuals with EDs (Thompson-Brenner et al., 2012), however, this research has focused exclusively on AN and BN. Given that the majority of help-seeking patients with BED are also obese, the stigma associated with obesity (‘weight bias’) is likely an important consideration in the treatment of patients with BED. Previous research has suggested that some medical practitioners are not immune from endorsing weight bias (Swift et al., 2013; Puhl et al., 2014). Indeed, medical practitioners reported having less respect for their patients as their BMI increases (Huizinga et al., 2009), believing treatments to be futile, and working with obese patients unfulfilling (Bocquier et al., 2005; Puhl and Heuer, 2009). However, to date, the intersection between the ED and obesity literature and clinical outcomes for patients with comorbid BED and obesity have not been investigated.

This study aimed to establish a baseline of Australian healthcare practitioners’ MHL of BED and attitudes toward weight, as a first step to understanding strengths and limitations in the current diagnosis and treatment of BED, particularly where BED is comorbid with obesity. As a common feature of BED (Montano et al., 2016), comorbid obesity was included to determine whether its presence masked the actual ED diagnosis when presenting to a frontline practitioner.

The specific aims were: (1) To explore MHL relating to BED with comorbid obesity, as compared with MHL relating to obesity only; and (2) To explore a baseline of weight bias amongst Australian healthcare professionals. A secondary aim was to compare weight bias amongst Australian healthcare professionals to previously published international data on weight bias amongst trainee or registered medical professionals (e.g., Berryman et al., 2006; Puhl et al., 2009).

## Materials and Methods

### Participants

Participants were Australian frontline healthcare professionals including GPs/physicians, psychiatrists, nurses, and dieticians. Recruitment was predominantly completed via an advertisement containing a broad description of the study and a link to the online survey, which was featured on the website, social media pages and/or electronic newsletter of 15 professional medical and allied health associations and academic institutions (e.g., Royal Australian College of General Practitioners, Eating Disorders Collaboration of Australia, Deakin University; please email the corresponding author for further details). Participants were also invited to provide the email addresses of colleagues who may wish to participate in the study. All participants were entered into the draw to win one of two double pass cinema tickets. A total of 279 participants commenced the online survey but 104 participants were excluded due to insufficient data on key study variables, leaving a final sample of 175 participants. Based on power of 0.80 and alpha set at 0.05 (two-tailed), this sample size was adequately powered to detect a small effect size (Cohen’s *d* > 0.38) corresponding with what Ferguson (2009) classified as ‘minimal effect size representing a practically significant effect for social science data.’

Participants (*n* = 175) were aged between 21 and 80 years old (*M* = 37.13, *SD* = 12.69), and tended to be GPs/physicians (48.5%), clinical dieticians (21.7%), or medical students (9.7%), with small numbers of registrars, nurses, psychologists, and psychiatrists. Most participants were female (85.7%), Caucasian (84%), and in a relationship (77.6%). The mean BMI was 23.75 (*SD* = 4.16). The majority of participants (73.8%) had a BMI in the ‘healthy range’ (BMI = 18.5-24.99), although 0.6% were classified as ‘underweight’ (BMI < 18.5), 18.6% were ‘pre-obese’ (BMI = 25-29.99), and 7% were ‘obese’ (BMI > 30). Ethical approval for the study was obtained from a university in Melbourne.

### Design and Procedure

The study involved an anonymous online questionnaire in which participants were randomly assigned to one of two case vignettes and asked to respond to self-report MHL questions related to diagnostic criteria, physical complications, and treatment options for comorbid BED and obesity (BED/obesity; experimental condition) or obesity (obesity-only; control condition). Participants were also presented with questions assessing attitudes toward weight (weight bias) and sociodemographics. The measures of MHL were adapted to the diagnoses in the two survey conditions, while the remaining section (weight bias) remained the same for both conditions. The total time commitment was approximately 15 min.

#### Demographics

Demographic questions enquired about participants’ age, gender, ethnicity, weight (kg), height (cm), occupational status and medical specialty. BMI was calculated based on self-reported height and weight.

#### MHL Relating to BED/Obesity – Case Vignettes

The format of the MHL measure was adapted from Currin et al. (2007, 2009) and Jones et al. (2013). Participants were presented with a case vignette, and were then required to indicate diagnoses from a selection of multiple choice response options. The case vignettes were adapted from Ebneter and Latner (2013), and involved only minor text alterations between conditions. In the BED/obesity condition, the case vignette involved a hypothetical patient who met the diagnostic criteria for comorbid BED and obesity. In the obesity-only condition, the hypothetical patient met the diagnostic criteria for obesity with no binge eating behaviors (Appendix A).

#### MHL Relating to BED/Obesity – Diagnostic Criteria, Physical Complications, and Treatment Options

Participants were then asked multiple-choice questions relating to diagnostic criteria, physical complications, and treatment options for BED/obesity. Participants were able to select multiple responses for each question. Each correct response was awarded one point; incorrect responses were not penalized.

(a) Diagnosis: Six options for diagnostic criteria were provided in each condition: four responses were correct in the BED/obesity condition (two distractor items), and one response was correct in the obesity-only condition (five distractor items). Criteria for BED were drawn from DSM-5 (APA, 2013), and from the Australian national medical guidelines for obesity (NHMRC, 2013).

(b) Physical complications: for BED and obesity were drawn from a number of publications (e.g., Sheehan and Herman, 2015; Mitchell, 2016). Ten of the 20 physical complications presented across both conditions were correct responses for both BED/obesity and obesity (a copy of the full list of physical complications including the corresponding references can be requested from the corresponding author).

(c) Treatment options: for BED and obesity were derived from an evidence-based review created by the National Eating Disorders Collaboration (NEDC, 2010) and National Medical Guidelines for Obesity (NHMRC, 2013). Six treatment options from a possible 14 were correct in each condition.

With the exception of changing ‘BED’ to ‘obesity,’ the same set of questions for diagnostic criteria, physical complications, and treatment recommendations were used in each condition.

#### Attitudes toward Weight (Weight Bias)

Weight bias was explored using the ‘Fat Phobia scale, short form’ (Bacon et al., 2001), a questionnaire containing 14 word-pair items (e.g., lazy, active). Both survey conditions used the same measure, with identical wording. Responses were made on a five-point Likert scale to indicate the word from each pair that participants believed best described individuals who are obese, with a score of ‘one’ indicating strong endorsement of the first word, and ‘five’ indicating strong endorsement of the second. The ‘short form’ was derived from a 50-item Fat Phobia Scale and focused on one factor measured in the original scale: ‘undisciplined, inactive and unappealing’ (Bacon et al., 2001). Overall weight bias scores were allocated by calculating the mean across items for each participant. Scores of > 2.5 indicated more ‘negative attitudes’ toward obese individuals. The ‘Fat Phobia scale, short form’ has previously demonstrated good correlation with the original long form (*r* = 0.82-0.90; Bacon et al., 2001), and had excellent reliability in the present study (Cronbach’s α = 0.85).

### Data Analysis

Data were analyzed using SPSS version 23 (IBM Corp, 2014). Missing data were excluded pair-wise from the relevant analyses as they represented less than 1% of overall data.

Descriptive statistics were calculated and *t*-tests (continuous measures) and Chi-square (categorical measures) analyses were used to determine if demographic, MHL, or weight bias differences existed between the BED/obesity and obesity-only groups. Independent samples *t*-tests were run to individually compare the mean weight bias score of the overall sample in the present study to the mean weight bias score recorded in six international studies that sampled trainee or registered healthcare professionals and used the Fat Phobia Scale, short form (Bacon et al., 2001) to probe weight bias (Poon and Tarrant, 2009; Puhl et al., 2009, 2014; Hayran et al., 2013; Swift et al., 2013; Bacardí-Gascón et al., 2015). The *t*-tests were conducted using GraphPad Software Inc. (2016) by entering the mean weight bias score, standard deviation (SD), and sample size into the software, and selecting the Welch test option to control for unequal group variances in these *t*-tests.

## Results

### Sociodemographics

As outlined in **Table 1**, no significant demographic differences were found between participants in the BED/obesity or obesity-only condition, with the exception of profession; χ^2^ = 22.45, *p* = 0.03, Cramer’s *V* = 0.36.

**Table 1:** Sociodemographics across the BED/obesity and the obesity-only groups.

		Experimental (BED/obesity) *n* = 79	Control (obesity-only) *n* = 96			
					
	Overall *n* (%)	*n* (%)	*n* (%)	χ^2^	*p*	*Phi* or *V*	
Gender						
Male	25 (14.3)	10 (12.7)	15 (15.6)	0.31	0.58	0.04
Female	150 (85.7)	69 (87.3)	81 (84.4)			
Ethnicity						
Caucasian	147 (84.0)	65 (82.3)	82 (85.4)	1.78^∗^	0.89	0.10
Eastern Asian	14 (8.0)	7 (8.9)	7 (7.3)			
Western Asian	1 (0.6)	0 (0.0)	1 (1.0)			
Middle Eastern	3 (1.7)	2 (2.5)	1 (1.0)			
Other	10 (5.7)	5 (6.3)	5 (5.2)			
Marital status						
Single	30 (17.1)	13 (16.5)	17 (17.7)	4.07	0.57	0.15
Married	74 (42.3)	29 (36.7)	45 (46.9)			
Divorced or separated	4 (2.3)	1 (1.3)	3 (3.1)			
In a relationship living together	46 (26.3)	24 (30.4)	22 (22.9)			
In a relationship not living together	15 (8.6)	9 (11.4)	6 (6.3)			
Other	5 (2.9)	2 (2.5)	3 (3.1)			
Profession				22.45	0.03	0.36
GP	13 (7.4)	1 (1.3)	12 (12.5)			
Physician	73 (41.7)	37 (46.8)	36 (37.5)			
Medical student or registrar	28 (16)	10 (12.6)	18 (18.75)			
Psychiatrist	2 (1.1)	2 (2.5)	0 (0.0)			
Nurse	9 (5.1)	6 (7.6)	3 (3.1)			
Dietitian	38 (21.7)	16 (20.3)	22 (22.9)			
Allied Health	12 (6.8)	6 (7.6)	6 (6.25)			

*‘Allied Health’ refers to other healthcare practitioners with a specialized (non-medical) training. ^∗^Fisher’s exact test used as at least one cell had a minimum expected count less than 5. *Phi* values are given as a measure of effect size for 2 × 2 chi square tests and Cramer’s *V* for all other analyses*.

### Baseline of MHL Relating to BED and Obesity

#### Diagnoses Indicated in Response to Case Vignettes

Diagnostic knowledge was assessed according to responses to the case vignettes and one question that required participants to indicate diagnostic criteria for BED/obesity. As outlined in **Table 2**, most participants in the BED/obesity condition correctly diagnosed the hypothetical patient with BED, however, only a quarter correctly diagnosed comorbid obesity. By comparison, over three quarters of the obesity-only group correctly recognized that the patient met diagnostic criteria for obesity. Chi-square tests for independence indicated that a significantly greater proportion of participants in the obesity-only condition diagnosed obesity as compared to the BED/obesity condition (χ^2^ = 44.54, *p* < 0.001, φ = 0.50), despite both hypothetical patients meeting criteria for obesity. A greater proportion of participants in the BED/obesity condition diagnosed BED as compared to the obesity-only condition (χ^2^ = 88.47, *p* < 0.001, φ = 0.71); **Table 2**.

**Table 2:** Diagnosis and diagnostic criteria indicated for each case vignette across each condition.

	BED/obesity *n* = 79	Obesity-only *n* = 96			
				
	*n* (%)	*n* (%)	χ^2^	*p*	*Phi*
Diagnosis					
Obsessive Compulsive Disorder (*n* = 0)	0 (0.0)	0 (0.0)	–		
Major Depressive Disorder (*n* = 3)	2 (3.0)	1 (1.0)	0.57	0.59	0.06
Bulimia Nervosa (*n* = 3)	3 (3.8)	0 (0.0)	3.71	0.09	0.15
Binge Eating Disorder (*n* = 76)	**65 (82.3)**	11 (11.5)	88.47	<0.001	0.71
Anorexia Nervosa (*n* = 2)	0 (0.0)	0 (0.0)	–		
Generalized Anxiety Disorder (*n* = 0)	0 (0.0)	2 (2.1)	1.67	0.50	0.10
Obesity (*n* = 95)	**21 (26.6)**	**74 (77.1)**	44.54	<0.001	0.50
Specific Phobia (*n* = 0)	0 (0.0)	0 (0.0)	–		
None of the above (*n* = 17)	3 (3.8)	14 (14.6)	5.75	0.02	0.18
Diagnostic Criteria					
Recurrent episodes of binge eating where a binge episode is characterized by eating within a discrete period of time (*n* = 61)	**61 (77.2)**	1 (1.0)	109.92	<0.001	0.79
A sense of lack of control over eating during the period of the binge (*n* = 76)	**73 (92.4)**	3 (3.1)	140.60	<0.001	0.90
Feeling disgusted with oneself, depressed, or very guilty after a binge (*n* = 55)	**52 (65.8)**	3 (3.1)	79.05	<0.001	0.67
BMI of 30 or above (*n* = 147)	8 (10.1)	**95 (99.0)**	141.22	<0.001	0.90
Over-valuation of weight and shape (*n* = 12)	9 (11.4)	3 (3.1)	4.64	0.03	0.16
Recurrent episodes of binge eating on average, at least once a week for 3 months (*n* = 65)	**64 (81.0)**	1 (1.0)	118.71	<0.001	0.82

*Bolded text indicates correct responses for each condition. Dash (–) indicates analysis not reported due to violations of assumptions of ‘minimum expected cell frequency.’*

Approximately half (49.4%) of the participants in the BED/obesity condition endorsed all four diagnostic criteria for BED, 29.1% identified three correct criteria, 11.4% identified two, and 8.9% identified one criteria (*M* = 3.14, *SD* = 1.03). The majority of the participants in the obesity-only condition endorsed the sole diagnostic criterion for obesity.

#### Physical Complications

Significant differences in the identification of physical complications associated with BED and obesity were seen across multiple-choice items, as indicated in **Table 3**. Chi-square tests for independence revealed that participants in the obesity-only condition correctly identified eight of the ten physical complications common to both obesity and BED significantly more often than participants in the BED/obesity condition did for BED.

**Table 3:** Physical complications endorsed by participants for each condition.

	BED/obesity participants who indicated physical complication associated with BED *n* = 79	Obesity-only participants who indicated physical complication associated with obesity *n* = 96			
				
Physical Complication	*n* (%)	*n* (%)	χ^2^	*p*	*Phi*
Electrolyte Imbalances**** (*n* = 20)	17 (21.5)	3 (3.1)	14.49	<0.001	0.29
Autoimmune disorders**** (*n* = 11)	4 (5.1)	7 (7.3)	0.37	0.76	0.05
Bradycardia (*n* = 1)	1 (1.3)	0 (0.0)	1.22	0.45	0.08
Hypoglycemia**** (*n* = 6)	2 (2.5)	4 (4.2)	36.70	<0.001	0.46
High blood pressure**** (*n* = 142)	**48 (60.8)**	**94 (97.9)**	39.10	<0.001	0.47
Osteoporosis**** (*n* = 15)	5 (6.3)	10 (10.4)	0.92	0.49	0.07
High cholesterol (*n* = 145)	**54 (68.4)**	**91 (94.8)**	21.33	<0.001	0.35
Chronic Kidney Problems of Kidney Failure (*n* = 41)	**10 (12.7)**	**31 (32.3)**	8.25	<0.05	0.22
Osteoarthritis (*n* = 94)	**19 (24.1)**	**75 (78.1)**	50.97	<0.001	0.54
Type II diabetes (*n* = 158)	**62 (78.5)**	**96 (100)**	20.49	<0.001	0.34
Stroke (*n* = 73)	**21 (26.6)**	**52 (54.2)**	13.56	<0.001	0.28
Irregular Menstrual Cycle (*n* = 95)	**41 (51.9)**	**54 (56.3)**	0.33	0.65	0.04
Skin Disorders (*n* = 68)	28 (35.4)	40 (41.7)	0.71	0.44	0.06
Tinnitus (*n* = 1)	0 (0.0)	1 (1.0)	0.83	1.00	0.06
Dental Erosion (*n* = 24)	15 (19.0)	9 (9.5)	3.38	0.078	0.14
Cardiovascular diseases (*n* = 136)	**43 (54.4)**	**93 (96.9)**	45.08	<0.001	0.51
Sleep apnea (*n* = 129)	**39 (49.4)**	**90 (93.8)**	44.06	<0.001	0.51
Polycystic ovary syndrome (*n* = 115)	**34 (43.0)**	**81 (84.4)**	32.87	<0.001	0.43

*Bolded text denotes correct responses for each condition*.

#### Knowledge of Treatment Options

Treatment recommendations endorsed by participants in each condition are presented in **Table 4**. The majority of participants in the BED/obesity condition correctly identified CBT for the treatment of BED, although fewer recognized other effective options such as interpersonal therapy. A Chi-square test for independence indicated that a significantly greater proportion of participants in the BED/obesity condition correctly selected CBT as a recommended treatment for BED as compared with participants who indicated that it was appropriate for obesity; χ^2^ = 20.06, *p* < 0.001, φ = 0.34. A significantly greater proportion of participants in the obesity-only condition correctly indicated that interpersonal therapy was a recommended treatment for obesity; χ^2^ = 8.48, *p* = 0.005, φ = 0.22.

**Table 4:** Treatment recommendations endorsed by participants for each condition.

	BED/obesity participants who endorsed treatment option *n* = 79	Obesity-only participants who endorsed treatment option *n* = 96			
				
Treatment	*n* (%)	*n* (%)	χ^2^	*p*	*Phi*
Psychodynamic Therapy	**22 (27.8)**	14 (14.6)	4.67	0.04	0.16
Behavioral Weight Loss	23 (29.1)	**85 (88.5)**	64.78	<0.001	0.61
Selective Serotonin Reuptake Inhibitors (SSRIs)	**30 (38)**	1 (1)	40.56	<0.001	0.48
Tricyclic Antidepressants	3 (1.3)	0 (0)	3.71	0.09	0.15
Anticonvulsant Medication	**1 (1.3)**	1 (1)	0.02	1.00	0.01
Cognitive Analytic Therapy (CAT)	7 (8.9)	6 (6.3)	0.43	0.57	0.05
Dialectic Behavioral Therapy (DBT)	**14 (17.7)**	6 (6.3)	5.63	0.03	0.18
Cognitive Behavioral Therapy (CBT)	**69 (87.3)**	**54 (56.3)**	20.06	<0.001	0.34
Eye movement desensitization and reprocessing	2 (2.5)	0 (0)	–		
Short Term Benzodiazepines	0 (0)	0 (0)	–		
Interpersonal Psychotherapy	**24 (30.4)**	**12 (12.5)**	8.48	0.01	0.22
Very low energy diets	3 (3.8)	**37 (38.5)**	38.50	<0.001	0.47
Weight loss medication	2 (2.5)	**27 (28.1)**	20.53	<0.001	0.34
Bariatric Surgery	5 (6.3)	**51 (53.1)**	43.61	<0.001	0.50

*Bolded text denotes correct responses for each condition. Dash (–) indicates analysis not reported due to violation of assumption of ‘minimum expected cell frequency.’*

### Assessing the Baseline of Weight Bias amongst Australian Healthcare Professionals

Significant differences in weight bias scores existed between groups such that the BED/obesity condition (*M* = 2.75, *SD* = 0.53) endorsed lower levels of weight bias as compared to the obesity-only condition (*M* = 2.98, *SD* = 0.67), *t*(171) = 6.44, *p* = 0.012, Cohen’s *d* = 0.38. Mean weight bias scores from both conditions were larger than 2.5 and thus indicative of ‘negative attitudes’ (Puhl et al., 2014). Subsequent analyses revealed that the mean weight bias score from the present study (*M* = 2.87, *SD* = 0.63) was significantly lower (i.e., less biased) when compared using individual *t*-tests with six international studies; **Table 5**.

**Table 5:** Independent samples *t*-tests to compare the mean weight bias score from the present study with previous international research.

Study	*n*	*M*	*SD*	*T*	*p*	CI		Cohen’s *d*
						LL	UL	
Bacardí-Gascón et al., 2015	634	3.45	0.69	10.56	<0.001	-0.69	-0.47	0.89
Hayran et al., 2013	305	3.57	0.69	11.31	<0.001	-0.82	-0.58	1.06
Puhl et al., 2009	182	3.70	0.51	13.65	<0.001	-0.95	-0.71	1.44
Puhl et al., 2014	329	3.16	0.47	5.35	<0.001	-0.40	-0.18	0.52
Poon and Tarrant, 2009	352	3.53	0.47	12.27	<0.001	-0.77	-0.55	1.19
Swift et al., 2013	1130	3.80	0.50	18.64	<0.001	-1.03	-0.83	1.63

*Each of the independent samples *t*-tests in the table above represents a comparison between the current data and the study listed. The present study had an *n* = 175, mean weight bias score = 2.87 (*SD* = 0.63)*.

## Discussion

This research aimed to examine Australian healthcare practitioner MHL and weight-related attitudes toward BED with comorbid obesity in comparison to obesity alone. Differences were revealed in MHL between participants in the two study conditions. The majority of participants correctly diagnosed the hypothetical patient with the primary presenting disorder in each condition. However, only a quarter of participants in the BED/obesity condition correctly diagnosed comorbid obesity. Participants in this condition also demonstrated significantly lower knowledge of diagnostic criteria and physical complications associated with BED as compared with knowledge of obesity in the obesity-only condition. Participants in both groups demonstrated limited knowledge of evidence-based treatment options. Finally, participants across both conditions endorsed weight bias scores that were indicative of negative attitudes toward obesity, however, they did so at levels that were significantly lower than healthcare professionals in previous studies (e.g., Hayran et al., 2013; Bacardí-Gascón et al., 2015). Each of these findings will be considered in the subsequent sections.

### MHL Relating to BED/Obesity Compared with Obesity

The majority of healthcare professionals accurately diagnosed the primary presenting issue in each condition with: BED in the BED/obesity condition and obesity in the obesity-only condition. This is a particularly impressive result given that BED was only recently added as a formal diagnosis to DSM-5 (APA, 2013), thus allowing healthcare practitioners a shorter period of time to gain relevant knowledge and experience with the disorder as compared with other EDs.

In contrast, the majority of participants in the BED/obesity condition did not recognize that their hypothetical patient also met diagnostic criteria for obesity. This difference existed despite the BMI of the hypothetical patients remaining the same across conditions (BMI = 35). The reluctance of participants to identify comorbid BED and obesity is consistent with data from Crow et al. (2004), who found that 41.6% of primary care physicians never assessed for binge eating amongst obese patients, and 42.8% believed that binge eating occurred in less than 20% of their obese patients. It is possible that healthcare professionals tend to underestimate the prevalence of comorbid BED and obesity presentations, and may be reluctant to assess for comorbid conditions once a primary diagnosis has been made. This reluctance may have clinical implications; as the majority of help-seeking individuals with BED are obese, successful diagnosis is likely to require an understanding of this common co-presentation (Montano et al., 2016). Moreover, in comparison with obesity alone, comorbid BED and obesity is associated with higher risk of poorer mental and physical health outcomes, and warrants close monitoring and specialized treatment (NEDC, 2015). It has also previously been noted that treatments for obesity and BED can be at odds with one another as the underlying motivation for behavioral weight loss (i.e., a social desirability framework; lose weight to be healthy and attractive) could potentially be harmful if applied to a patient with an ED who, by definition, experiences body shame, self-esteem issues, and internalization of the thin sociocultural ideal (NEDC, 2010). However, this is a controversial point requiring further exploration, as some studies have also demonstrated that treatments such as behavioral weight loss do not appear to exacerbate BED symptomatology, and may even be as efficacious as CBT in reducing eating-related psychopathology and occurrences of binge eating (Munsch et al., 2007; Grilo et al., 2011). Nevertheless, healthcare professionals’ reluctance or difficulty in diagnosing comorbid BED and obesity is of considerable concern.

In relation to diagnostic knowledge of BED and obesity, the majority of participants in the obesity-only condition recognized the sole criterion for obesity. In comparison, approximately half of the BED/obesity group correctly identified all four diagnostic criteria for BED. Limited literature exists to interpret these data in terms of the precise level of knowledge that might be considered ‘good enough’ in relation to BED. However, the rates of identifying individual criteria compared favorably with previous studies which required medical professionals identify individual diagnostic criteria for AN and BN (Currin et al., 2007, 2009; Jones et al., 2013). Healthcare professionals’ identification of individual diagnostic criteria for BED and obesity was thus considered a strong facet of MHL within the present sample.

### Knowledge of Physical Complications Related to BED/Obesity Compared with Obesity

A key finding from the current study was that healthcare professionals demonstrated a relatively limited understanding of physical complications associated with BED as compared with obesity. Nine of the possible physical complications presented to participants in both conditions were correct for both BED and obesity. Participants in the obesity-only condition identified eight of these nine complications for obesity, significantly more than participants in the BED/obesity condition did for BED. This result is concerning given that patients with EDs frequently present to healthcare professionals with physical complaints associated with their primary diagnosis, and diagnosis of BED often relies on the identification of associated medical complications (Mond et al., 2007; NEDC, 2010).

### Knowledge of Recommended Treatment Options

Consistent with the different treatment recommendations for BED and obesity (NEDC, 2010; NHMRC, 2013), participants in the BED/obesity condition correctly identified CBT as a recommended treatment more frequently than participants in the obesity-only condition, whilst participants in the obesity-only condition recommended behavioral weight loss more than the BED/obesity condition. Although these findings suggest that healthcare professionals have an accurate knowledge of the recommended treatment for BED, the majority of participants did not tend to select additional treatments that have been shown to have comparable efficacy. For example, Interpersonal Psychotherapy was endorsed for obesity more commonly than it was selected for BED despite displaying a comparable efficacy with CBT in the treatment of BED (NEDC, 2010). The data from the present study would suggest that healthcare professionals readily recognize CBT to treat BED to the exclusion of other potentially efficacious treatment options.

### Weight Bias

As predicted, the overall sample demonstrated weight bias toward obese individuals (Puhl et al., 2014). Surprisingly, participants in the BED/obesity condition held significantly less negative attitudes toward weight compared with participants in the obesity-only condition. Subsequent analysis suggested that although the mean weight bias endorsed by the entire sample in the present study was indicative of ‘negative attitudes’ toward individuals who are obese (Puhl et al., 2014), it was significantly lower than weight bias observed amongst a range of international samples of trainee or registered healthcare professionals (e.g., Puhl and Heuer, 2009; Swift et al., 2013).

The comparatively low levels of weight bias reported in the present study compared with previous findings are promising, yet challenging to interpret. Although we cannot unequivocally rule out the possibility that the nature of the study resulted in participants with lower weight bias opting to participate, this explanation seems unlikely, as the study was advertised in very broad terms and made no mention of attitudes to weight or weight-related disorders. The observed differences might be accounted for by a priming effect. In the comparative studies (e.g., Poon and Tarrant, 2009), participants first completed other measures relating to implicit and explicit weight bias which may have primed stereotyped attitudes, whilst in the present study, participants first responded to case vignettes and knowledge questions which might have primed a professional/medical mindset. Alternatively, levels of weight bias may have been related to the body weight characteristics of the current sample; a high proportion of the sample (more than 20%) were classified as pre-obese or obese, and previous research has identified an inverse relationship between one’s own body weight and level of weight bias (Schwartz et al., 2006). Finally, it is possible that the lower levels of weight bias were indicative of a real reduction in stereotypical attitudes toward overweight and obese persons. However, although lower than previous reported findings, mean weight bias scores across both conditions remained suggestive of ‘negative attitudes’ toward obesity (Bacon et al., 2001), and thus stereotyped attitudes are an important issue in the treatment of obese patients.

### Strengths and Limitations

The study has a number of strengths, including that is it the first to explore healthcare practitioner knowledge and attitudes in relation to BED in an Australian context, and the first to assess practitioner MHL of BED since the disorder was classified as a formal diagnosis in DSM-V (APA, 2013). In addition, the sample included a range of healthcare practitioners, enhancing generalizability across professions likely to encounter individuals with BED. The use of the case vignette methodology was a further strength of the study, as experimental manipulation enabled differences in clinical presentation to be examined in ways that would be difficult in alternative designs. However, it is possible that specific features of the vignettes influenced the findings in unanticipated ways. For example, the behavior of the hypothetical patient in the BED/obesity vignette was explicitly described as binge eating. Given that a well-documented challenge in diagnosing BED is determining the difference between overeating and binge eating (Grilo et al., 2001), it is possible that the specific use of the term in the vignette increased the ability of the participants to accurately diagnose BED. An additional potential limitation of the materials was that the 10 diagnostic options presented to participants following the vignettes were displayed in a fixed order in the online survey, with BED appearing three places above obesity in the 10 item list. Although further investigation is required to determine whether presentation order had a significant influence on results, it is possible that randomizing the diagnostic choices to appear in different locations in the list may have led to an increase in comorbid diagnoses of BED and obesity in the BED/obesity condition. Several further limitations must be acknowledged. The use of surveys provided no opportunity to clarify or seek elaboration on participants’ clinical decision-making processes. Future studies may wish to incorporate participant interviews in addition to survey responses to gain a more nuanced understanding of clinical diagnostic and treatment decisions in relation to EDs. The obtainable sample size was limited by challenges in recruitment, including lengthy application processes to recruit through large associations and limited direct access to association members. As a result, the final sample in each condition was relatively small and could not be analyzed according to profession. Finally, future studies may wish to include a third vignette condition describing a BED patient with normal weight to further tease out the differences in practitioner MHL for BED as opposed to obesity, and may also consider including male hypothetical patients in vignettes to investigate whether the gender of the patient impacts practitioner attitudes.

### Clinical Implications

The overarching aim of the present study was to explore barriers to the diagnosis and treatment of EDs that may occur specifically at the level of the healthcare practitioner, which corresponds to tasks three and four of the help-seeking pathway depicted in **Figure 1A**. To place our findings into context, **Figure 1B** builds on this model of help-seeking by identifying the potential barriers to successfully completing these tasks that were supported or extended by the present study.

Healthcare professionals’ endorsement of somewhat negative attitudes toward weight likely has a clinical implication at task three, which relies heavily on a positive interaction between patient and practitioner. This stage requires that the practitioner create a safe space for the disclosure of ED symptomology and the patient displays a readiness to disclose in this space. Implicit or explicit weight bias might be readily perceived by a patient who endorses internalized weight bias, and may thus impact their willingness to engage with the practitioner.

In regards to task four of the help-seeking pathway, our findings extend previous knowledge by suggesting that due to deficits in MHL, healthcare professionals might face specific difficulties in recognizing common BED presentations, including comorbid BED and obesity and associated physical complications. Results indicated that healthcare professionals may also rely on limited treatment options, which may be problematic if patients do not respond favorably to a CBT-based treatment approach.

The findings thus suggest that clinical training in EDs, and BED in particular, needs not only to focus on improving diagnostic and treatment knowledge, but also to address healthcare practitioners’ pre-existing attitudes toward eating and weight in order to facilitate successful progress through the help-seeking pathway.

## Conclusion

While the majority of Australian healthcare practitioners in this study accurately diagnosed hypothetical patients with a primary diagnosis of BED or obesity, they tended not to diagnose comorbid BED and obesity. Deficits in practitioner knowledge were observed in the identification of physical complications commonly associated with BED as compared with obesity, which may impact the rates of diagnosis of BED as the majority of patients with EDs present to medical settings with a medical complication as the primary presenting issue (Mond et al., 2007). Knowledge of treatment options appeared to be accurate, but narrow. Participants across both conditions endorsed a level of weight bias suggestive of negative attitudes toward patients who are obese, but at a lower level than previous research (e.g., Puhl et al., 2014). Further research is required to model the way in which the BED help-seeking literature fits together. The proposed model (**Figure 1**) provides an attempt to conceptualize the data to date, however, the literature is lacking in longitudinal studies that track patients with EDs from initiation of a help-seeking process to the end of treatment to determine the factors that impact help-seeking, diagnosis and effective treatment.

## Ethics Statement

This study was carried out in accordance with the recommendations of the HESC ethics committee at the University of Melbourne with written informed consent from all subjects. All subjects gave written informed consent in accordance with the Declaration of Helsinki. The protocol was approved by the HESC.

## Author Contributions

BC, KB, and IK, drafted the manuscript and conceptualized the aims and hypotheses. MF-T conducted the analyses. BC and IK set up data collection. All authors provided feedback on different versions of the manuscripts. All authors read and approved the final manuscript and are accountable for all aspects of the work in ensuring that questions related to the accuracy of any part of the work are appropriately investigated.

## Conflict of Interest Statement

The authors declare that the research was conducted in the absence of any commercial or financial relationships that could be construed as a potential conflict of interest.
