# Supplementary material for: Australian Healthcare Professionals’ Knowledge of and Attitudes toward Binge Eating Disorder
Source: Front Psychol. 2017 Aug 7;8:1291. doi: 10.3389/fpsyg.2017.01291 (PMC5545598; doi:10.3389/fpsyg.2017.01291)
Supplement: Supplementary file 1 [file Data_Sheet_1.docx]

**Appendix A**

**Case Vignette used for BED/obesity condition where hypothetical patient, ‘Emily’ meets diagnostic criteria for comorbid BED and obesity**

Emily is a 19-year-old second-year art student. Emily has been overweight since she was an adolescent but in recent years this has increased to the point where her BMI is 35. Over the years Emily has tried a number of diets and healthy eating plans; however, she has never stayed with the recommendations for very long. Emily lives by herself and often feels lonely; to counteract these feelings Emily likes to ‘‘treat’’ herself with luxurious foods such as chocolate and cheesecake. Emily’s diet is generally regular, with three meals a day, and it contains a wide variety of foods. When Emily gets home from school she often goes to the fridge for a small snack; however, Emily finds that after eating the snack she is unable to control or stop her eating and continues to eat a large amount of food for a period of approximately two hours. She may binge eat, for example, on two slices of cheesecake, a bag of cookies, a jam sandwich and three glasses of milk in one sitting. Later in the evening she will eat dinner and sometimes she loses control with this also and eats the extra helping that she was planning to save for the next day. Emily feels uncomfortably full later at night and guilt and sadness after she has eaten like this, however she does not engage in any compensatory behaviors (e.g. purging). She has engaged in this binge-eating cycle twice a week for the past six months. Emily is very distressed by her eating behaviors, however has never disclosed this issue to anyone.

**Case vignette used for control condition whereby hypothetical patient, ‘Valerie’ met diagnostic criteria for obesity only**

Valerie is a 19-year-old second-year art student. Valerie has had a BMI of 25 since she was an adolescent but in recent years this has increased to a BMI of 35. Over the years Valerie has tried a number of diets and healthy eating plans; however, she has never stayed with the recommendations for very long and she feels helpless about her prospects of reducing her weight. Exercising is much harder as well. She used to love to play tennis, but no longer. Part of the problem is that she ends up eating the kind of foods her boyfriend and friends eat. These include pastas, sugary cereals, cookies and other unhealthy and fattening foods. None of her friends are overweight, nor is her boyfriend, who also loves pasta. Subsequently, Valerie is constantly surrounded by fattening food and finds it very hard to resist. She also tends to eat unhealthy foods under times of stress and when she is upset. This is when her diets tend to fail.
